# Bilateral renal angiomyolipoma in a patient with tuberous sclerosis treated with resection of one kidney and transarterial embolization of other kidney using CT during selective arteriography: a case report

**DOI:** 10.4076/1757-1626-2-6351

**Published:** 2009-07-31

**Authors:** Yoshiaki Katada, Isao Umehara, Takemasa Ohki, Mitsuhiro Kishino, Hitoshi Shibuya

**Affiliations:** 1 Department of Radiology, Saitama Red Cross Hospital8-3-33, Kamiochiai, Chuo-ku, Saitama-shi, Saitama 338-8553Japan; 2 Department of Radiology, Asahi General Hospital1326, i, Asahi-shi, Chiba 289-2511Japan; 3 Department of Urology, Narita Red Cross Hospital90-1, iida-cho, Narita-shi, Chiba 286-8523Japan; 4 Department of Radiology, Faculty of Medicine, Tokyo Medical and Dental University1-5-45, Yushima, Bunkyo-ku, Tokyo 113-8519Japan

## Abstract

Renal AML complicating tuberous sclerosis shows a rapid growth and its rupture is frequently associated with hemorrhagic shock as a result of profuse retroperitoneal bleeding, necessitating an aggressive therapeutic approach. This report describes the long-term clinical progress of 28 year-old woman with tuberous sclerosis with a ruptured giant AML that underwent unilateral nephrectomy, who has been followed up after treatment with concomitant application of computed tomography during selective arteriography to conserve the remaining normal renal parenchyma, and in whom the need for initiation of dialysis has been successfully avoided, with conspicuous reduction of the tumor size.

## Introduction

AML of the kidney is a benign neoplastic lesion that is composed of vascular and smooth muscle components and adipose tissue, and has come to be recognized as a rather common complication of tuberous sclerosis [1,2]. AML complicating tuberous sclerosis shows a rapid growth and its rupture is frequently associated with hemorrhagic shock as a result of profuse retroperitoneal bleeding, necessitating an aggressive therapeutic approach [1-3].

## Case presentation

The patient, a 28-year-old Japanese woman, had been under periodic follow-up since her childhood for tuberous sclerosis, and underwent right nephrectomy for AML at the age of 17; she continued to be under follow-up for immediate detection of AML developing in the remaining left kidney. During the course of follow-up, the patient developed sudden abdominal pain and needed emergency hospitalization because of decreased serum Hb level. Laboratory examination at admission revealed a serum Hb level of 7.3 g/dL and serum Cre of 0.9 mg/dL.

**Figure 1.**
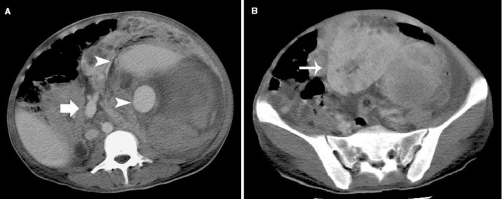
CT scans on admission. Abdominal CT demonstrated a fairly large-sized AML in the left retroperitoneum and containing a huge aneurysm-like hemorrhage (arrow heads). The left renal artery was dilated to nearly the size of the aorta (thick arrow). **(B)** A part of the normal renal parenchyma was identified (thin arrow).

Abdominal CT revealed a fairly large-sized tumorous lesion situated primarily in the left retroperitoneum and containing a huge aneurysm-like hemorrhage. The left renal artery was dilated to nearly the size of the aorta (Figure 1A). While a part of the normal kidney parenchyma was identified (Figure 1B), most of the remaining organ was occupied by the AML and constituents of the hematoma secondary to rupture, that conspicuously impinged on and displaced the abdominal viscera (Figure 1A, Figure 1B).

**Figure 2.**
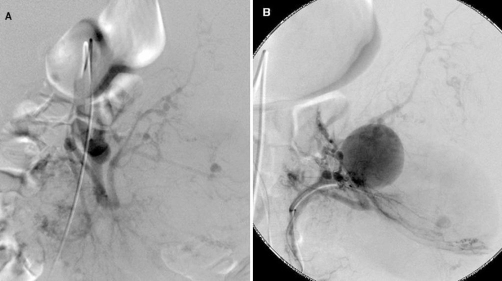
Left renal arteriogram. **(A)** The mass was poorly visualized due to the conspicuous increase of blood flow, despite injection of contrast medium even at the rate of 7 mL/sec (total dose: 35 mL). **(B)** An aneurysm-like hemorrhage was visualized. A pseudoaneurysm and tumor blushes are also noted.

**Figure 3.**
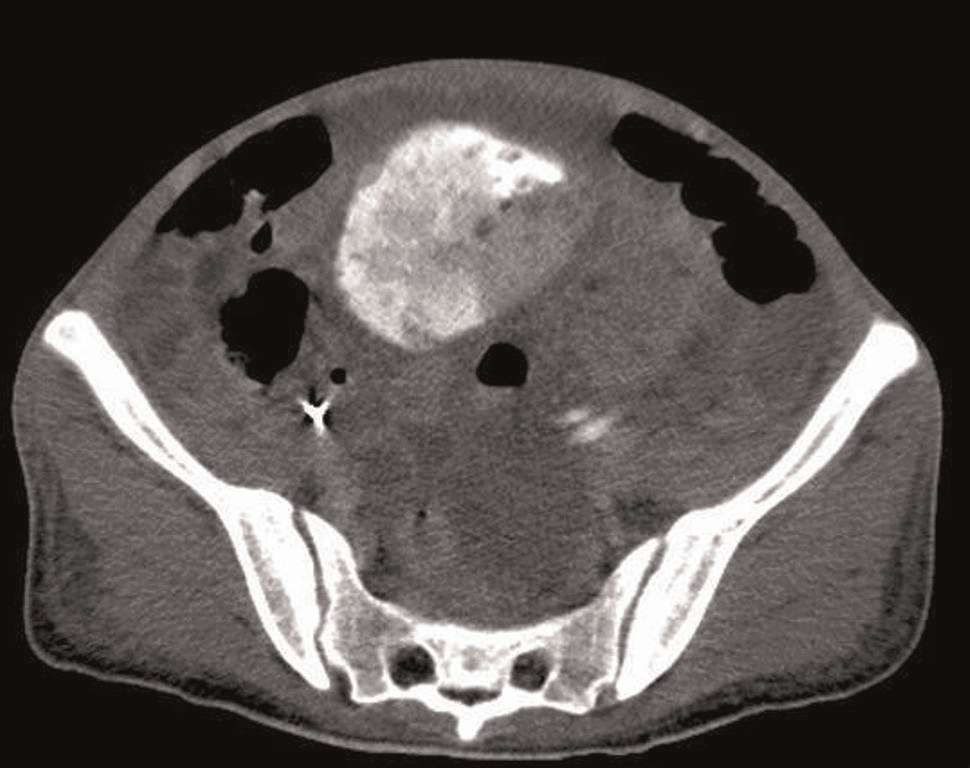
CT during selective arteriography. Most of the normal renal parenchyma can be visualized, allowing precise identification of the feeding blood vessels of the normal kidney, although there are filling defects in a part of the normal kidney.

**Figure 4.**
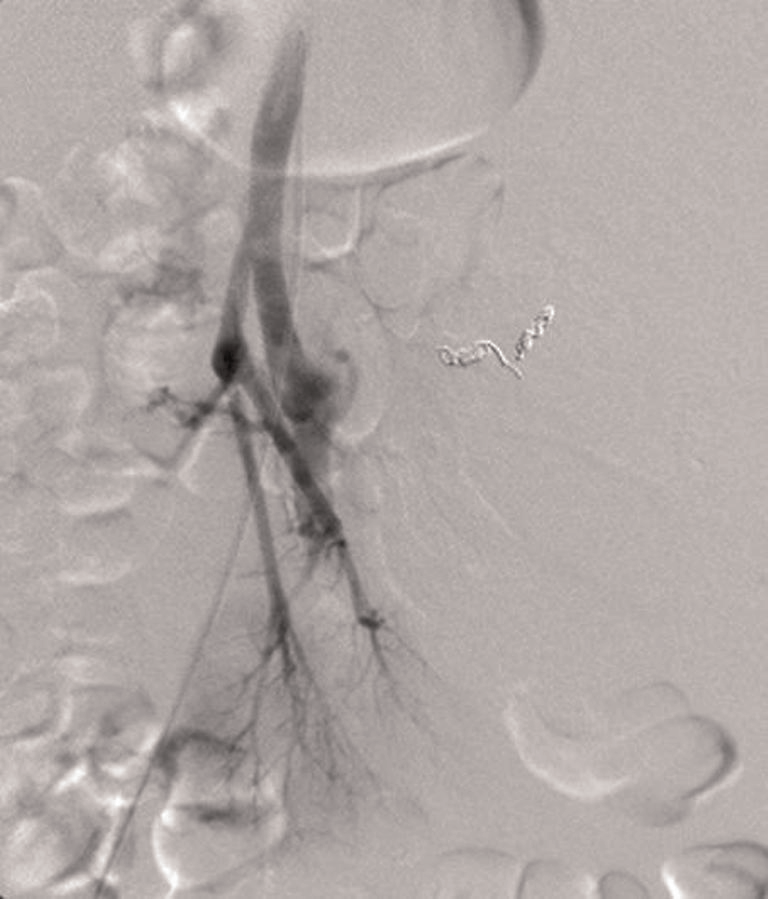
Left renal arteriogram after the embolization procedure angiography performed after the embolization procedure demonstrated satisfactory visualization of the normal renal parenchyma and the remaining embolized tumor vasculature.

An abdominal angiography revealed marked distension of the left renal artery, and it was difficult to visualize the mass clearly and to accurately delineate the vascular supplies to the normal kidney parenchyma, even by left renal arteriography (infusion rate, 7 mL/sec; total dose of the contrast medium, 35 mL) (Figure 2A). Based on these findings, we carried out embolization of the aneurysm-like bleeding site with metallic coils (Figure 2B) and of the rest of the bleeding sites with gelatin sponge particles upon identifying the blood vessels supplying the normal renal parenchyma, with concomitant CTSA (Figure 3). Angiography performed after the embolization procedure demonstrated satisfactory visualization of the normal renal parenchyma and the remaining embolized tumor vasculature (Figure 4).

**Figure 5.**
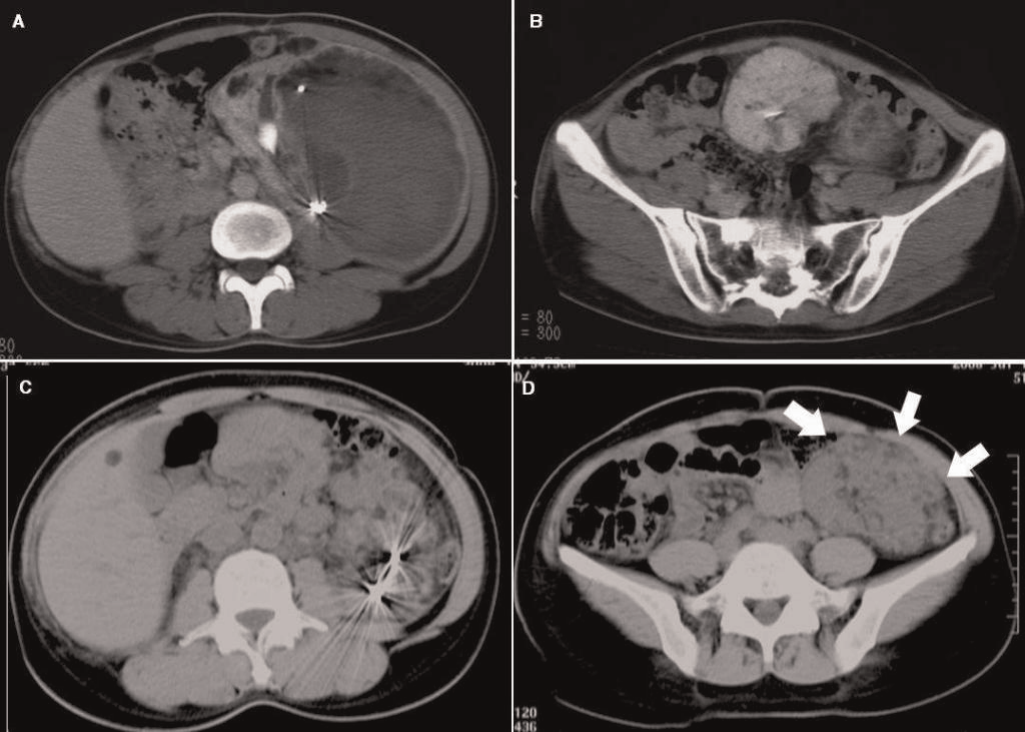
CT scans at half a year **(A-B)** and 5 years after **(C-D)** the treatment **(A-D)** Gradual diminution of the retroperitoneal hematoma and angiomuscular components of the tumor. The conserved renal parenchyma is satisfactorily visualized. **(C-D)** CT performed 5 years after the treatment demonstrated disappearance of the hematoma and angiomuscular components, but enlargement of the angiomyolipoma developing from the conserved kidney (thick arrows).

At half a year and about 5 years after the embolization, CT scans showed marked contraction in the size of the tumor and hematoma, with no evidence of any fresh hemorrhage (Figure 5a, 5b, 5c, and 5d). The CT obtained at 5 years post embolization, nevertheless, demonstrated an increase in the size of the AML arising from the left kidney (Figure 5c, 5d). In regard to the renal function, the serum Cre level was 1.5 mg/dL at the time of discharge and remained practically constant thereafter, except for one instance of worsening when the patient developed septicemia of uterine origin during the 4th year post operation. The serum Cre level has remained in the 3 to 3.5 mg/dL range, and the patient has not needed initiation of dialysis therapy.

## Discussion

Tuberous sclerosis is frequently complicated by AML, which is known to occur more frequently as multiple lesions and to grow to larger sizes as compared with idiopathic AML. It has also been documented that AML associated with tuberous sclerosis shows a high likelihood of rupture during the clinical course, with resultant hemorrhagic shock secondary to hematuria and retroperitoneal bleeding [1-10]. In regard to transcatheter arterial embolization for the treatment of AML, Moorhead et al. [4] reported the results of embolization with gelatin sponge particles in 1977; thereafter, several investigators have reported the results of the procedure in both patients with ruptured and unruptured AML [4-8,10]. Currently, embolization represents the therapy of first choice in a significant proportion of the cases.

In the present case, the attempt to delineate the whole mass by angiography was virtually unsuccessful even after injection of 35 mL of contrast medium at the rate of 7 mL/sec. Therefore, we employed CTSA, which enabled efficient differentiation between the feeding arteries to the tumor and the normal renal vasculature with the use of a smaller volume of contrast medium early after the start of treatment, and thereby, efficient embolization therapy. Marked amelioration of the abdominal compressive symptoms was also anticipated with the expected contraction of the retroperitoneal hematoma with the ruptured AML and reduction in the diameter of the tumor. We used metallic coils in this case, but the use of metallic coils calls for discretion, that is, their use should be limited solely to embolization at the site of rupture of an aneurysm, because they may not only give rise to the formation of collateral circuits to the tumor, but also cause revascularization of the metallic coil and pose a hindrance at the next intervention.

Our present patient has been under periodic follow-up for over 5 years; a long-sustained contraction of the hematoma and angiomyomatous components of the tumor has been evident from the time the treatment was administered until date. Although introduction of dialysis may hardly be avoidable with possible enlargement of the AML arising from the left kidney in the future [7], its avoidance for over 5 years is considered beneficial for patients.

## Conclusion

Selective embolization is recommended even in cases with rupture of a giant AML developing in a unilateral residual kidney because embolization of the tumor vasculature and the attempt to conserve normal renal parenchyma to the greatest extent possible provides not only hemostasis, but also results in reduction of the tumor diameter and conservation of renal function. In the present case, the adoption of this therapeutic strategy with concomitant CT during selective arteriography yielded favorable outcomes both in the short-term and in the long-term.

## Abbreviations

AML: angiomyolipoma
Cre: creatinine
CT: computed tomography
CTSA: CT during selective arteriography
Hb: hemoglobin
